# Advanced spectroscopy-based phenotyping offers a potential solution to the ash dieback epidemic

**DOI:** 10.1038/s41598-018-35770-0

**Published:** 2018-11-28

**Authors:** Caterina Villari, Arnaud Dowkiw, Rasmus Enderle, Marjan Ghasemkhani, Thomas Kirisits, Erik D. Kjær, Diana Marčiulynienė, Lea V. McKinney, Berthold Metzler, Facundo Muñoz, Lene R. Nielsen, Alfas Pliūra, Lars-Göran Stener, Vytautas Suchockas, Luis Rodriguez-Saona, Pierluigi Bonello, Michelle Cleary

**Affiliations:** 1 Department of Plant Pathology, The Ohio State University, 201 Kottman Hall, 2021 Coffey Road, 43210 Columbus Ohio, United States; 2 Warnell School of Forestry & Natural Resources, University of Georgia, 180 E Green Street, 30602 Athens, Georgia United States; 3 Institut National de la Recherche Agronomique (INRA), UMR 0588 BioForA, 2163 Avenue de la Pomme de Pin, 45075 Orléans Cedex 2, France; 4 Forest Research Institute Baden-Wuerttemberg, Department Forest Protection, Wonnhaldestrasse 4, 79100 Freiburg, Germany; 5 Institute for Plant Protection in Horticulture and Forests, Federal Research Centre for Cultivated Plants (Julius Kühn Institute), Messeweg 11/12, 38104 Braunschweig, Germany; 6 Swedish University of Agricultural Sciences (SLU), Southern Swedish Forest Research Centre, Sundsvägen 3, 23053 Alnarp, Sweden; 7 Institute of Forest Entomology, Forest Pathology and Forest Protection (IFFF), Department of Forest and Soil Sciences, University of Natural Resources and Life Sciences, Vienna (BOKU), Peter-Jordan-Straße 82, 1190 Vienna, Austria; 8 Department of Geosciences and Natural Resource Management, University of Copenhagen, Rolighedsvej 23, 1958 Frederiksberg C., Denmark; 9 Lithuanian Research Centre for Agriculture and Forestry, Institute of Forestry, Liepu 1, LT53101 Girionys, Kaunas district Lithuania; 10 SKOGFORSK - The Forest Research Institute, Ekebo 2250, 26890 Svalöv, Sweden; 11 Department of Food Science and Technology, The Ohio State University, Parker Food Science and Technology, 2015 Fyffe Road, 43210 Columbus Ohio, United States

## Abstract

Natural and urban forests worldwide are increasingly threatened by global change resulting from human-mediated factors, including invasions by lethal exotic pathogens. Ash dieback (ADB), incited by the alien invasive fungus *Hymenoscyphus fraxineus*, has caused large-scale population decline of European ash (*Fraxinus excelsior*) across Europe, and is threatening to functionally extirpate this tree species. Genetically controlled host resistance is a key element to ensure European ash survival and to restore this keystone species where it has been decimated. We know that a low proportion of the natural population of European ash expresses heritable, quantitative resistance that is stable across environments. To exploit this resource for breeding and restoration efforts, tools that allow for effective and efficient, rapid identification and deployment of superior genotypes are now sorely needed. Here we show that Fourier-transform infrared (FT-IR) spectroscopy of phenolic extracts from uninfected bark tissue, coupled with a model based on soft independent modelling of class analogy (SIMCA), can robustly discriminate between ADB-resistant and susceptible European ash. The model was validated with populations of European ash grown across six European countries. Our work demonstrates that this approach can efficiently advance the effort to save such fundamental forest resource in Europe and elsewhere.

## Introduction

Invasive, alien tree pathogens threaten biodiversity, ecosystem services, and commercial forestry on a global scale^1–3^. Such bioinvasions are increasing at an unprecedented rate, in part because of higher global connectivity^4^, which facilitates unintended long-distance movement of pathogens into regions outside their historical distribution range^5^, and because of climate change, which causes host maladaptation^6^ and emergence of new pathogens through alteration of host-microbe interactions^7^. These disturbances have the potential to cause massive and irreversible damage^8^ by eliminating keystone tree species in many areas of the world, and permanently altering trophic structures, nutrient dynamics^1^ and primary productivity^2^ of forest communities. Such wide ranging ecological impacts can compromise the maintenance of ecosystem services upon which humans rely, including those associated with reduced incidence of human morbidities^9^.

Genetically controlled resistance is crucial to successfully manage natural and urban forests for resilience against alien invasive pathogens^10^. Molecular markers are invaluable to enhance screening for resistance, and can be applied to practical breeding programs with high precision and reductions in cost and time. However, marker-assisted selection has not been used for genetic improvement of forest trees to the same extent as agricultural crops^11^. Indeed, efficient marker development requires accurate field testing, which in trees can be very slow due to the long time lag intrinsic in symptom development^12^. This lack of efficient tools for rapid resistance phenotyping significantly hinders our ability to screen natural populations for conventional breeding of trees. Our ability to readily and reliably detect superior genotypes could enhance the success of current restoration efforts, or protect trees from logging or other activities associated with forest and urban landscape management, in support of *in situ* conservation.

We hypothesized that disease-resistant genotypes can be identified through chemometrics using vibrational spectroscopy, which is based on the absorption of infrared radiation resulting from fundamental molecular (bond) vibrations. This technique can rapidly fingerprint a wide range of biological samples using multivariate statistical classification models that identify and delineate target classes^13–15^. Considering that plant resistance against pests and pathogens essentially relies on host chemistry^16^, vibrational spectroscopy-based techniques hold vast potential in distinguishing between plant chemical phenotypes (chemotypes) that are genetically and epigenetically driven, and vary in disease susceptibility. Among these techniques, Fourier-transform infrared (FT-IR) spectroscopy has so far shown promising results in applications involving forest trees^13^, including delineating resistance to invasive pathogens such as *Ophiostoma novo-ulmi*, causal agent of Dutch elm disease^17,18^, and *Phytophthora ramorum*, causal agent of sudden oak death^19^. In the latter case, the technique was able to successfully model tree resistance prior to infection.

At present, there is rising concern that European ash (*Fraxinus excelsior*), an important keystone species in natural plant communities protected under EU legislation^20^, may be functionally extirpated from European forests by the alien invasive fungus, *Hymenoscyphus fraxineus*, causal agent of ash dieback (ADB)^21,22^. A low proportion of the natural population of European ash expresses heritable, quantitative resistance that is stable across environments^23–25^, and genomic solutions to uncover mechanisms associated with disease resistance have been explored as a means to accelerate breeding of trees with resistance against ADB^26,27^. In a recent paper, Sollars *et al*.^28^ found an association between disease susceptibility and the levels of two putative iridoid glycosides in the leaves of *F*. *excelsior*, which suggests that chemotypes associated with this pathosystem can be targeted and tested using FT-IR spectroscopy and chemometric models. Consequently, our goal was to determine the feasibility and efficacy of FT-IR to phenotype European ash for resistance to ADB.

## Results and Discussion

**Figure 1:**
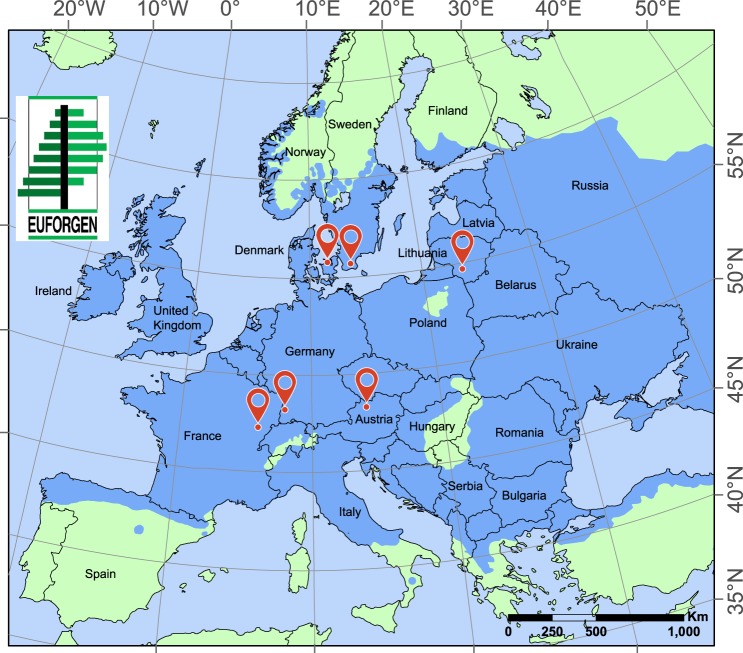
Map of the sampling sites. Uninfected bark and leaf samples were collected from a total of 76 *Fraxinus excelsior* genotypes of known susceptibility to *Hymenoscyphus fraxineus* in six European countries: Austria, Denmark, France, Germany, Lithuania, and Sweden. Sampling sites (red mapping pins) are overlaid on the natural distribution map of *F*. *excelsior* (sky-blue) (EUFORGEN 2009, www.euforgen.org.).

We analysed the Fourier transform mid-infrared spectral region^14,29^ of phenolic extracts^16^ of uninfected tissue samples from 76 different genotypes collected across six European countries (Austria, Denmark, France, Germany, Lithuania and Sweden) (Fig. 1) that were previously phenotyped as having either low, intermediate or high susceptibility to *H*. *fraxineus*^24,25,30–36^ (Table 1). We targeted phenolics due to the established role of this class of secondary metabolites in general plant defense^16,37^, and the successful prior phenotyping efforts using this technique in other pathosystems^19^. Samples comprised both leaves and twig bark, which included a thin outer bark (<1 mm thick), cortex, secondary phloem, and cambial tissues. To test the robustness and reliability of our approach, we ensured broad heterogeneity of the samples within each tissue type by sampling trees at different developmental stages and environmental conditions across locations. Up to three ramets per genotype were sampled, for a total of 134 trees across the six countries (Table 1). To be practically useful, chemotypes should be associated with constitutive composition and levels of specialized phytochemicals. Therefore, it was critical to ensure that *H*. *fraxineus* was not present in plant tissues. Despite observations at the time of sample collection of some ripe apothecia on pseudosclerotial leaf rachises in the litter of moist microsites at the Austrian location, all samples from all locations were confirmed free of the pathogen via PCR^38^. All trees may have harboured infections in other parts of the tree, prior to our observations in this study, and thus we cannot exclude possible systemic effects of these other infections on the chemotypes. However, previous work in the coast live oak – sudden oak death pathosystem has shown that healthy tissues sampled away from active cankers were chemically indistinguishable from healthy tissues taken from asymptomatic and presumably uninfected trees^39^.

**Table 1:** Detailed information on the *Fraxinus excelsior* genotypes analysed in the study.

Country^*^	Location	Trial Details					Date of sample collection in 2015	Number of genotypes sampled per susceptibility class (for clonal trials, the number of ramets per genotype is given in parentheses)		
		Type of genetic trial	Coordinates	Elevation (m asl)	Year established	Spacing of trees (m)		Sus.	Int.	Res.
Austria^31^^,^^36^^**^	Feldkirchen an der Donau	Clonal seed orchard	48°20′11.4″ N 14°02′53.2″ E	264	1993	7.5 × 8.6	9 June	7 (2)		7 (2)
Denmark^32^	Tuse næs	Clonal seed orchard	55°45′ 58.0″ N 11°42′ 47.4″ E	22	1998	3.0 × 6.0	2, 4 June	3 (3)	2 (3)	3 (3)
France^24^	Devecey	Provenance and progeny trial	47°19′31.5″ N 06°01′54.1″ E	250	1995	4.0 × 4.0	18 June	7	3	7
Germany^35^	Weisweil	Provenance trial	48°11′29.7″ N 07°42′02.5″ E	173	2005	2.0 × 2.0	19 May	5		5
Lithuania^34^	Sasnava	Clonal collection	54°37′32.1″ N 23°33′55.5″ E	100	2012	6.0 × 5.4	2 June	2 (3), 2 (2)	2 (2)	3 (3), 1 (2), 1 (1)
Sweden^30^	Snogeholm	Clonal seed orchard	55°32′33.8″ N 13°42′22.7′′ E	50	1992	3.5 × 3.5	28 May	3 (1), 1 (2)	5 (2)	5 (2), 2 (2)
Total number of genotypes								30	12	34
Total number of trees								50	23	61

*For all countries except Lithuania, genotypes in each trial originated in the same country where the trial is located. Genotypes in the Lithuanian trial originated in the Czech Republic, Germany, Ireland and Lithuania.

**Literature reference number.

Sus., susceptible; Int., intermediate; Res., resistant.

**Figure 2:**
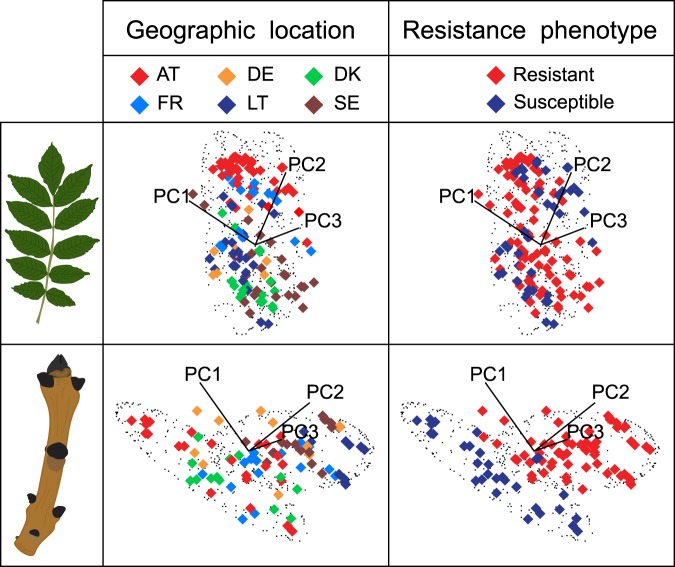
SIMCA 3D class projections. SIMCA 3D class projection plots for spectral data of *Fraxinus excelsior* leaf and twig bark tissue phenolic extracts analysed as a function of the resistance phenotype, but visualized according to either the sample geographic location or its resistance phenotype. Spectral data were pre-processed using the standard normal variate function and then smoothed and transformed into their second derivative. Two technical replicates were analysed separately. Clouds of black points indicate the 95% confidence interval for each class (i.e., resistance phenotypes) in each principal component direction (i.e., PC1, PC2 and PC3) projected into the three-factor principal component hyper-plane.

We processed FT-IR chemical fingerprints using a soft independent modelling of class analogy (SIMCA) chemometric approach to discriminate between different resistance phenotypes^13^. In order to resolve overlapping peaks, minimize background and improve the model predictions, spectral data were pre-processed via the standard normal variate function^40^, and smoothed and transformed into their second derivative using a Savitzky-Golay polynomial filter^41^. We initially analysed both leaf and twig bark tissues, for a total of 131 and 112 samples, respectively. Preliminary observations of the SIMCA 3D class projection plots of resistant and susceptible samples showed that geographic location of the trees strongly affected the chemistry of the leaves (Fig. 2). This difference may be attributable to a higher sensitivity of foliage to solar irradiation^42^, temperature^43^ and nutrient or water availability^44^ associated with the different latitudes, geographic locations, and microclimates. However, this effect was not evident in twig bark tissues (Fig. 2), therefore, we built the chemometric model using only the FT-IR spectra of the twig bark samples. We do not exclude the possibility that resistance is expressed at the leaf level^31^; rather, we suggest that environmental variation masked any possible chemical signature in the leaf tissues. Indeed, in previous studies^28^, leaf constitutive iridoid glycosides could discriminate among European ash resistance phenotypes. Secoiridoids have also been shown to be upregulated in the leaves of *F*. *excelsior* in response to treatment with a *H*. *fraxineus* toxin^45^.

To optimize the model, clustering patterns of the resistance phenotypes were sought in the SIMCA 3D class projection plot (Fig. 2), while spectral regions with the highest discriminating power were identified using SIMCA discriminating power plots and Coomans plots^46^. We used Coomans plots and 3D class projection plots also to identify outliers, which were removed from the model. After trimming the data in this way, the complete data set comprised a minimum of two and up to seven genotypes per susceptibility class per country, and up to three ramets per clone, for a total of 71 samples (42 resistant, 7 intermediate, and 22 susceptible). Of these, 75% of samples within each geographic location and susceptibility class were selected for the training data set to build our calibration model, while the remaining 25% were used as the testing data set for model validation^13^. Samples were randomly assigned to the groups; in doing so, 10 clones were represented by ramets in both the training and testing data sets, while eight clones were unique to the testing population. Therefore, the testing dataset verified the ability of the model to classify the resistance phenotype for both clones that were independent of the training population and those that were biological replicates of clones included in the training population. Incremental analysis of different models showed that inclusion of intermediate phenotypes in the calibration compromised the formulation and strength of the chemometric classification. This was to be expected, as SIMCA models work best when built on well-defined groups^13,19^. We hence decided to exclude intermediates from the training set and move all of them to the testing set.

**Figure 3:**
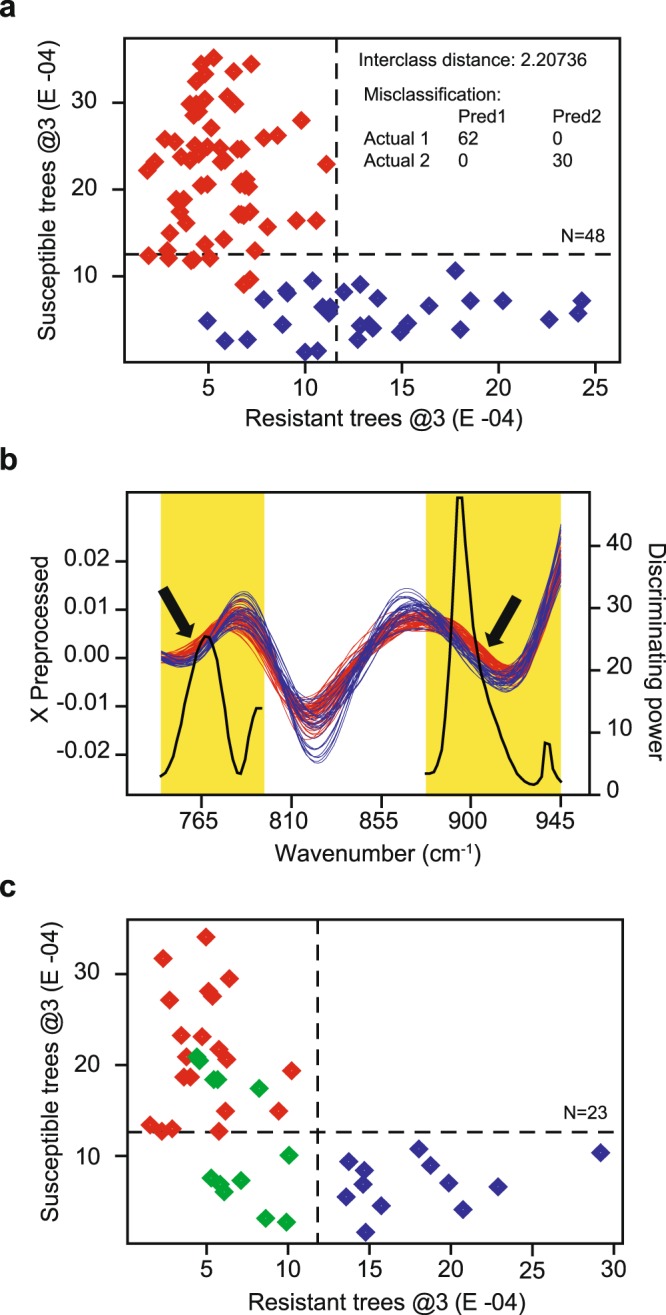
SIMCA Coomans plots and discriminating power plot. Panel a, SIMCA Coomans plot showing the relative, dimension-free distance between the samples of the training data set used to build the 3-factor (@3) calibration model designed to discriminate between ash dieback resistant (red diamonds) and susceptible (blue diamonds) *Fraxinus excelsior* trees. X-axis represents the distance from the resistant class, while y-axis represents the distance from the susceptible class. Two technical replicates were analysed separately, for a total of 92 spectra, corresponding to 48 biological replicates. Dashed lines indicate critical sample residual thresholds. Panel b, SIMCA discriminating power plot of the 3-factor calibration model. The discriminating power (black line) is overlaid on the second derivative, smoothed and standard normal variate transformed spectra. The SIMCA calibration model that best discriminated between resistant (red lines) and susceptible (blue lines) ash trees included spectral regions from ~748 to 798 cm^−1^ and from ~879 to 947 cm^−1^ wavenumber (highlighted in yellow). The black arrows point to regions of the spectra where the discrimination between the two resistance phenotypes is evident by visual inspection. Panel c, SIMCA Coomans plot showing the relative, dimension-free distance between the samples of the testing data set used to validate the 3-factor (@3) model. In addition to resistant (red diamonds) and susceptible (blue diamonds) trees, the testing data set included accessions of intermediate phenotype (green diamonds), based on field observations. Two technical replicates were analysed separately, for a total of 44 spectra, corresponding to 23 biological replicates randomly selected from each of the six European countries. Dashed lines indicate critical sample residual thresholds.

The SIMCA calibration model that best discriminated between resistant and susceptible ash trees was a 3-factor model (Fig. 3a) obtained by including spectral regions from ~748 to 798 cm^−1^ and from ~879 to 947 cm^−1^ wavenumber (Fig. 3b), which primarily correspond to the C−H wagging of substituted benzenes of aromatic compounds^47^. The highest discriminating power peak (discriminating power of 47.7) corresponded to ~895 cm^−1^ wavenumber, which may also correspond to the wagging of the hydrogen on the C-1 position of the cellulose glucose ring^48^. However, since the extraction protocol we adopted is highly specific for phenolic compounds^49^, the presence of polysaccharides in the analysed extracts is very unlikely, and the ~895 cm^−1^ wavenumber almost certainly corresponds to the C−H wagging of the aromatic hydrocarbon groups of phenols^47^. The second highest discriminating power peak (discriminating power of 25.4) corresponded to ~770 cm^−1^ wavenumber. Interclass distance between the groups was 2.2074, indicating good separation between the phenotypes, and 100% of extracts from both resistant and susceptible ash trees were correctly classified, showing high specificity of the model. The low number of factors included in the model (i.e., three) argues against model overfitting^50^, with factors 1–3 explaining 97.87%, 1.21% and 0.53%, respectively, of the variability in the susceptible class, and 97.10%, 1.42%, and 1.00%, respectively, of the variability in the resistant class.

Validation of the chemical marker-based statistical model is a crucial step to ensure accurate predictions and confirm the applicability of the model at a larger scale^13^. We validated our SIMCA model with the testing data set, and 100% of the ten resistant and six susceptible ash ramets randomly selected from each of the six European countries (including nine representing biological replicates of the clones included in the training data population) were correctly identified as belonging solely to their phenotype group (Fig. 3c). Four of the intermediate samples, corresponding to four different genotypes, were identified as potentially belonging to both classes, which correctly reflects their intermediate phenotype. However, the remaining three samples, corresponding to two different genotypes, were identified as belonging solely to the resistant group (Fig. 3c). One of the intermediate genotypes had different ramets that were classified in either groups (resistant and intermediate). Such overestimation of the resistant samples demonstrates that while the model may not be perfect with intermediates, it clearly separated the most susceptible samples from the rest. Furthermore, the overall accuracy of the model was 87%, which is rather impressive, given the vast heterogeneity of host genotype, developmental stage, and environmental conditions represented by the tested ash populations.

Our results show that European ash possesses readily exploitable levels of resistance that can be detected using FT-IR spectroscopy. This work represents a major advancement in the application of marker-assisted technology for tree breeding and offers a strong proof of concept of a novel solution in the fight against ADB. Compared to labor-intensive and time-consuming traditional tree phenotyping techniques based on artificial inoculations or natural infection assays, or even nascent nucleic acid-based phenotyping^51^, FT-IR spectroscopy can significantly accelerate the process of selecting resistant phenotypes and limit the need for growing out large segregating progenies as in conventional breeding programs. This technique can be applicable at a landscape level for screening large naïve populations for disease resistance, at dramatically reduced costs than traditional selection. With this rapid phenotyping approach, breeding for resistance, *ex situ* and *in situ* conservation, restoration, and long-term sustainable management of this threatened tree species becomes a feasible and realistic strategy to mitigate the ADB epidemic. This method can also pave the way for rapid progress in managing the global forest health crises resulting from many other chronic and emerging forest diseases where there is evidence of some level of resistance in the naïve host populations.

## Methods

### Plant material

Uninfected bark (including a thin outer bark (<1 mm thick), cortex, secondary phloem, and cambial tissues) and leaf samples were collected from a total of 76 *F*. *excelsior* genotypes with known susceptibility to *H*. *fraxineus* in six European countries: Austria, Denmark, France, Germany, Lithuania, and Sweden. Source material originated from genetic trials established as either clonal seed orchards^30–32,36^ or for testing ash provenance^35^ or progeny^24,25,33,34^ (Table 1). Sample collection was performed between 19 May and 18 June 2015. At each site, a minimum of three and up to seven genotypes were selected from the extreme ends of the spectrum of host susceptibility^19^ (low vs high), while a minimum of two and up to five genotypes of intermediate resistance were selected when available (i.e., from Denmark, France, Lithuania, and Sweden). All selections were based on a relative measurement and ranking of disease severity at the crown level (i.e. dieback intensity) as determined in previous assessments^24,30–32,34–36^. In the case of clonal trials, up to three ramets per clone were sampled, for a total of 134 trees (Table 1). High heterogeneity of tree developmental stage and environmental conditions was purposely included in order to test the robustness and broad applicability of the model. The current year’s shoots were harvested from each individual tree. Due to the occurrence of a late frost event in Lithuania, epicormic shoots instead of crown shoots were harvested from both resistant and susceptible genotypes. Similarly, epicormic shoots were the only ones present and suitable for harvesting from some of the susceptible genotypes across the different countries, which provided a further source of variation.

Leaves collected in the field were labelled according to country, trial, family, ramet (if pertinent) and susceptibility status, placed in a plastic bag and immediately stored on dry ice. Bark was either dissected from the internodes of the twig supporting the leaf rachis with a sterile razor blade or scalpel, and similarly labelled and stored on dry ice, or whole twigs were immediately frozen on dry ice in the field and tissues were dissected later in the lab. All samples were then transported or shipped frozen to the Swedish University of Agricultural Sciences in Alnarp, Sweden and stored at −20 °C until further processing.

### Sample processing and phenolic extraction and purification

To avoid any potential degradation or oxidation of the tissues, samples were constantly kept frozen at −20 °C or in liquid nitrogen during all processing steps preceding phenolic extraction. Bark and leaf tissues were finely ground in liquid nitrogen and 200 ± 1 mg aliquots of either tissue type were placed in individual 2 ml microcentrifuge tubes.

Phenolic compounds were extracted and purified following the protocol described by Wrolstad *et al*.^49^, with modifications. Ground tissue was submerged in 700 µl of 70% HPLC-grade acetone (Sigma-Aldrich Sweden AB, Stockholm, Sweden) in Milli-Q (Merck Chemicals and Life Science AB, Solna, Sweden) purified water (v/v). Samples were vortexed at maximum speed for 10 sec and then subjected to sonication for 30 min at room temperature, followed by centrifugation at 1,600 rcf for 8 min. The supernatant was transferred to a new 2 ml tube and twice the volume of HPLC-grade chloroform (Sigma-Aldrich Sweden AB) was added. Vials were inverted by hand two times and then vortexed at maximum speed for 15 sec prior to centrifugation at 10,000 rcf for 2 minutes at 10 °C. Working on ice, the aqueous phase, containing the phenolic compounds, was collected and transferred to a new 2 ml screw-cap tube with O-ring seal. Samples were then lyophilized and shipped at room temperature to the Department of Plant Pathology, The Ohio State University. Working on ice, lyophilized crude extracts were resuspended in 1 ml of Milli-Q (Millipore, Bedford, Massachusetts, USA) purified water and sonicated for 30 min in water and ice to allow complete solution of the pellet prior to the phenolic solid-phase purification step. Phenolic compounds were purified on an Xpertek SPE Snap Cap 300 mg C_18_ silica cartridge (Cobert Associates, Inc., Saint Louis, Missouri, USA). Cartridges were conditioned with HPLC-grade methanol (Fisher Scientific, Pittsburgh, Pennsylvania, USA) and equilibrated with Milli-Q water. After forcing extracts through the cartridge, cartridges were washed twice with Milli-Q water and then the adsorbed compounds were eluted in HPLC-grade methanol. Before use, extracts were evaporated to dryness in a Vacufuge vacuum concentrator (Eppendorf, Westbury, New York, USA) and re-dissolved in HPLC-grade methanol to a final concentration of 10 times the original one. Purified and concentrated phenolic extracts were stored at −20 °C until further analysis.

### Molecular detection of *Hymenoscyphus fraxineus* in leaves and bark

The absence of *H*. *fraxineus* from all analysed leaves and twig bark tissues at the time of sampling was verified by PCR using species-specific ITS primers^38^. DNA was extracted in 2 ml screw cap tubes containing 30 mg of homogenized tissue using the E.Z.N.A. SP Plant DNA Kit (Omega Bio-tek, Doraville, Georgia, USA), according to manufacturer’s instructions. Amplifications were performed in 10 µl reaction volumes containing 0.05 μl of Taq Polymerase (5 u/μl); 1.0 μl of 10x Buffer; 1.0 μl of dNTPs (2 mM); 0.15 μl of MgCl_2_ (25 mM); 0.2 μl of each primer *Chafra* F/*Chafra* R, 5.0 μl of sample DNA (0.5 ng/μl) and 2.4 μl of milli-Q water. Each PCR run included a no-template negative control of water, and DNA of *H*. *fraxineus* isolate nf4 collected in Sweden as positive control. The PCR cycling conditions included an initial denaturation step at 95 °C for 5 min followed by 35 amplification cycles of denaturation at 95 °C for 30 s, annealing at 56 °C for 30 s, and extension at 72 °C for 1 min. The reaction was terminated by an extension step at 72 °C for 7 min. PCR products were visualized by gel electrophoresis on a 1.5% agarose gel in TE buffer (Sigma-Aldrich Sweden AB) stained with GelRed™. The GeneRuler mix (Fermentas, Burlington, Ontario, Canada) was used as size standard.

### FT-IR analysis

FT-IR spectroscopy of the phenolic extracts was carried out on an Excalibur 3500GX FT-IR spectrometer (Digilab, Randolph, Massachusetts, USA), equipped with a potassium bromide beamsplitter and a MIRacle triple-bounce zinc selenide crystal (Pike Technologies, Madison, Wisconsin, USA) attenuated total reflectance (ATR) accessory. Either 6 µl or 5 µl of concentrated extracts were placed on the ATR crystal and vacuum dried for approximately 1 min or 40 sec for leaves and twig bark samples, respectively. Spectra were collected in the mid-infrared region, over a wavenumber range of 4000 to 700 cm^−1^. Resolution was set at 4 cm^−1^ and an interferogram of 64 scans was co-added for each sample. Two technical replicates were collected for each sample, and spectra were displayed in terms of absorbance using Win-IR Pro Software (Agilent Technologies, Santa Clara, California, USA)^19^. Total number of spectral data collected, net of any tissue processing losses, was 262 for leaf samples and 224 for twig bark tissues, corresponding to 131 and 112 samples, respectively.

We carried out SIMCA multivariate analysis of the spectral data using the chemometric modelling software Pirouette (v. 4.5, Infometrix Inc., Bothell, Washington, USA). SIMCA classification technique develops 3D principal components models for each training group (in this case resistant, intermediate and susceptible ash trees) and identifies the most important variables for the discrimination of groups, while preserving relevant information and reducing noise^52^. Spectral data were pre-processed via the standard normal variate function to remove multiplicative scatter and particle size interference^40^, and then smoothed and transformed into their second derivative using a 35-points Savitzky-Golay polynomial filter to increase the signal-to-noise ratio, minimize background and reduce overlapping bands^41^. Technical replicates were analysed separately^19^.

Preliminary analyses with the spectra of resistant and susceptible leaf samples clearly showed that clustering patterns of the SIMCA 3D class projection plot were mainly driven by the geographic location of the trees, while no pattern was associated with different resistance phenotypes (Fig. 2). Chemical composition of leaves is strongly affected by environmental factors such as nutrient and water availability^44^, low temperatures^43^ or solar irradiation^42^, and this high variability might have masked any potential chemical signal associated with resistance. We hence concluded that leaves were not ideal and were therefore excluded from further analysis. On the other hand, preliminary analyses with the spectra of resistant and susceptible twig bark samples showed that clustering patterns of the SIMCA 3D class projection plot were mainly driven by the resistance level of phenotypes, while geographic location did not show any strong patterns (Fig. 2).

Optimization of the SIMCA model was obtained by initially including the whole collected wavenumber range (4000 to 700 cm^−1^) of all 224 spectral data for twig bark tissues, and then progressively refining the model both by reducing the spectral range to those regions with the highest discriminating power (Fig. 3c), and by removing outliers. Each incrementally refined model was evaluated by observing clustering patterns of the resistance phenotypes in the 3D class projection and Coomans plots, and by evaluating the discriminating power plot^46^. Outliers were visually identified on the Coomans plot and 3D class projection plot. Based on their chemical signature, all nine Swedish and all six Lithuanian genotypes originally classified as intermediate and susceptible, based on field observations of the extent of dieback, were classified by the model as resistant and intermediate, respectively. This discrepancy in the classification, together with a general trend of overestimating resistance, may be attributable to some random variation among ramets, or most likely the use of slightly different parameters in the evaluation of susceptible phenotypes in Sweden^30^ and Lithuania^25^. The inclusion of uncertain phenotypes in both the training and testing sets would have compromised the strength of the chemometric model and its validation; thus intermediate and susceptible Swedish and Lithuanian genotypes were excluded from further analyses. Incremental refinement of the training models showed that, while overall clustering of the groups was not changing, the inclusion of intermediates significantly reduced the discriminating power. This is an expected outcome, as SIMCA models are known to work best when built on well-defined groups^13,19^ (in this case, the extreme ends of the spectrum of host susceptibility). Therefore, we decided to exclude intermediates from the training set and move them to the testing set instead. We opted for this approach, as we wanted to verify if the model was still able to correctly classify all intermediate samples included in the analyses. The total number of samples included in the training model, net of any trimming, was 48 (32 resistant and 16 susceptible) and 92 spectral data, while the testing data set comprised 10 resistant, seven intermediate and six susceptible samples, for a total of 23 samples and 44 spectral data. Model accuracy was calculated as the percentage of correctly identified samples relative to the total number of samples included in the testing data set.

## Data Availability

The datasets generated and analysed during the current study are available from the corresponding authors upon reasonable request.
